# (1*R*,2*S*,4*S*,4a*S*,8*S*,8a*S*)-4-Hy­droxy-8,8a-dimethyl-10-oxo-2,3,4,7,8,8a-hexa­hydro-1*H*-4a,1-(ep­oxy­methano)­naphthalen-2-yl acetate

**DOI:** 10.1107/S1600536813013524

**Published:** 2013-05-22

**Authors:** Ouassila Selaïmia-Ferdjani, Chahra Bidjou-Haiour, Aurelien Planchat, Muriel Pipelier

**Affiliations:** a Laboratoire de Synthèse Organique Modélisation et Optimisation des Procédés Chimiques, Université Badji-Mokhtar Annaba, BP12, 23000 Annaba, Algeria; b Université de Nantes, CNRS, Laboratoire CEISAM-UMR 6230, Faculté des Sciences et des Techniques, 2 rue de la Houssinière, BP 92208, 44322 Nantes Cedex 3, France

## Abstract

The title compound, C_15_H_20_O_5_, presents a bis­norsesquiterpene skeleton, with a *trans*-deca­line backbone constrained by the lactone bridge. The α-hy­droxy substituent and the methyl group belonging to the two deca­line rings are in axial positions, whereas the other methyl group and the acyl group occupy the sterically preferred equatorial positions. The mol­ecular structure is stabilized by an intra­molecular C—H⋯O hydrogen bond. In the crystal, mol­ecules are linked into chains along [010] by O—H⋯O hydrogen bonds

## Related literature

For the synthesis, see: Selaimia-Ferdjani *et al.* (2013 ▶). For the biological activity of the natural lactone Paralemnolide A analogue of the title compound, see: Wang *et al.* (2012 ▶) and of related nardosinane sesquiterpene derivatives, see: Bishara *et al.* (2008 ▶); Huang *et al.* (2011 ▶); Petit *et al.* (2004 ▶); Lu *et al.* (2011 ▶). For related nardosinane sesquiterpenes whose biological activity has not been investigated, see: El-Gamal *et al.* (2005 ▶); Huang *et al.* (2006 ▶); Wang & Duh (2007 ▶); Wang *et al.* (2010 ▶).
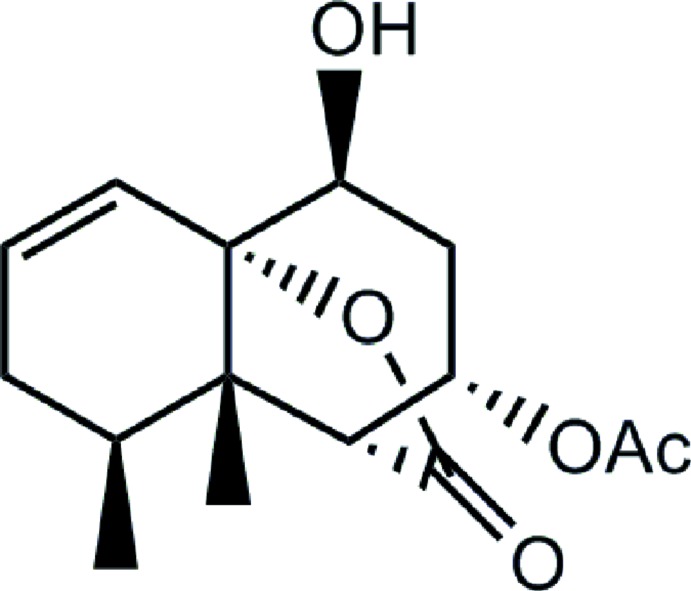


## Experimental

### 

#### Crystal data

C_15_H_20_O_5_
*M* *_r_* = 280.3
Monoclinic, 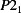

*a* = 10.3312 (10) Å
*b* = 7.1692 (8) Å
*c* = 10.8502 (6) Å
β = 115.958 (5)°
*V* = 722.56 (12) Å^3^
*Z* = 2
Mo *K*α radiation
μ = 0.10 mm^−1^
*T* = 293 K
0.48 × 0.42 × 0.30 mm

#### Data collection

Nonius KappaCCD diffractometer
Absorption correction: gaussian (*JANA2006*; Petříček *et al.*, 2006) ▶ *T*_min_ = 0.967, *T*_max_ = 0.972
12282 measured reflections
3328 independent reflections
2774 reflections with *I* > 2σ(*I*)
*R*_int_ = 0.049

#### Refinement

*R*[*F* ^2^ > 2σ(*F* ^2^)] = 0.050
*wR*(*F* ^2^) = 0.138
*S* = 1.84
3328 reflections
185 parameters
H atoms treated by a mixture of independent and constrained refinement
Δρ_max_ = 0.15 e Å^−3^
Δρ_min_ = −0.12 e Å^−3^

### 

Data collection: *COLLECT* (Nonius, 1998 ▶); cell refinement: *EVALCCD* (Duisenberg *et al.*, 2003 ▶); data reduction: *COLLECT* (Nonius, 1998 ▶); program(s) used to solve structure: *SIR2004* (Burla *et al.*, 2005 ▶); program(s) used to refine structure: *JANA2006* (Petříček *et al.*, 2006) ▶; molecular graphics: *Mercury* (Macrae *et al.*, 2006 ▶); software used to prepare material for publication: *JANA2006* ▶.

## Supplementary Material

Crystal structure: contains datablock(s) global, I. DOI: 10.1107/S1600536813013524/bt6907sup1.cif

Structure factors: contains datablock(s) I. DOI: 10.1107/S1600536813013524/bt6907Isup2.hkl

Additional supplementary materials:  crystallographic information; 3D view; checkCIF report

## Figures and Tables

**Table 1:** Hydrogen-bond geometry (Å, °)

*D*—H⋯*A*	*D*—H	H⋯*A*	*D*⋯*A*	*D*—H⋯*A*
C10—H3*c*10⋯O4	0.96	2.31	2.946 (4)	122.91
O4—H1⋯O9^i^	0.83 (5)	2.10 (4)	2.907 (2)	165 (4)

Symmetry code: (i) 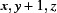.

**Fig. 1.**
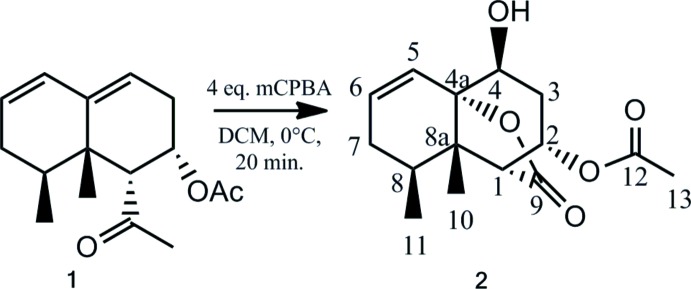
Synthetic scheme to prepare title compound.

**Fig. 2.**
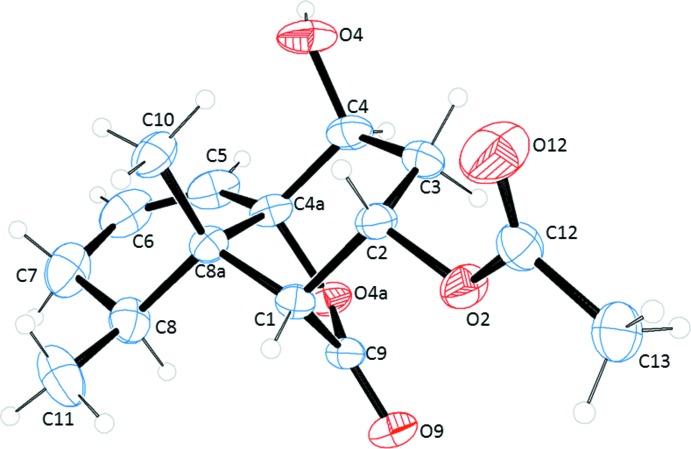
ORTEP drawing of the X-Ray crystallographic structure of the title molecule, with atom labeling. The displacement ellipsoids are drawn at the 30% probability level. H atoms are presented as small spheres of arbitrary radius

**Fig. 3.**
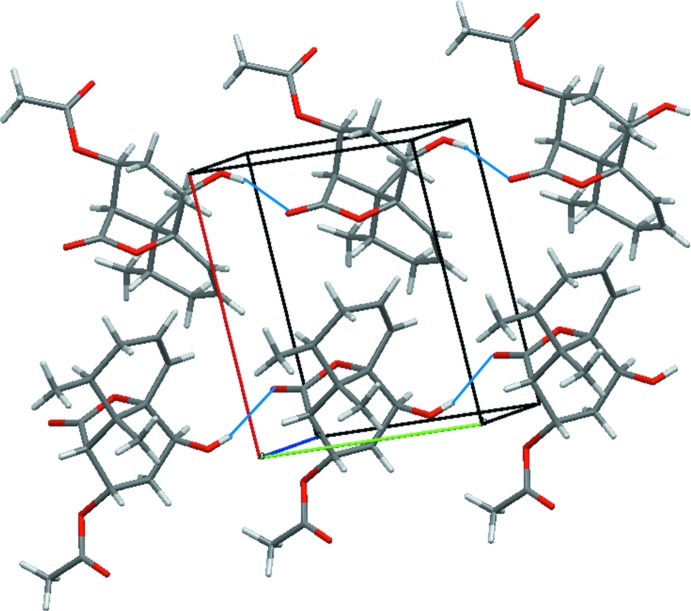
Portion of the crystal structure showing the packing of the H–bonded infinite chains of the title compound. The intermolecular O-H···O bonds are depicted by blue lines.

## supplementary crystallographic information

### Comment

Several nardosinane sesquiterpene derivatives, extracted from different soft corals (Lemnalia, Paralemnalia, Rhytisma and others), have already expressed promising biological properties (Bishara *et al.* 2008; Huang *et al.* 2011; Lu *et al*. 2011; Petit *et al.* 2004; Wang *et al.* 2012) while the potential of others remains to be explored (El-Gamal *et al.* 2005; Huang *et al.* 2006; Wang and Duh 2007; Wang *et al.* 2010). In our continuing interest in the total synthesis of biologically active compounds, we have recently proposed a synthetic strategy to access such sesquiterpene derivatives. In the course of the synthesis, the title compound appeared as an analogue structurally close to the natural lactone Paralemnolide A (Wang *et al.* 2012) which possesses cytotoxic activity. We report herein on the crystal structure of a new nardosinane sesquiterpene analogue.

The title compound presents a bisnorsesquiterpene skeleton, with a *trans*-decaline backbone constrained by the lactone bridge. The α-hydroxy substituent and the methyl group belonging to the two decaline rings are in an axial position, whereas the other methyl group and the acyl group occupy the sterically preferred equatorial position. In the crystal, the molecules form chains connected by O-H···O hydrogen bonds. The crystal structure is further stabilized by C-H···O contacts.

### Experimental

Title compound was synthesized according to the reported method (Selaimia-Ferdjani *et al.*, 2013): To a solution of diene 1 (100 mg, 0.38 mmol) in CH_2_Cl_2_ (2.5 ml) at 0°C was added *via* cannula a solution of mCPBA (350 mg, 0.38 mmol) in CH_2_Cl_2_ (5.0 ml). After 20 min, the reaction mixture was quenched with saturated NaHCO_3_ solution (10 ml). The aqueous layer was extracted with CH_2_Cl_2_ (3 *x* 25 ml) then the combined organic layers were washed with brine (50 ml), dried over MgSO_4_, filtered and concentrated under vacuum. The residue was purified by flash chromatography (eluant Petroleum Ether-EtOAc 100/0 to 7/3) to afford lactone 2 (title compound) (63 mg, 60%) as a solide. Crystals suitable for X-ray structure analysis (colorless crystals) were obtained by slow evaporation of a solution of the title compound in ethyl acetate/hexane (1:1, *v*/*v*) at room temperature. mp = 434 K; [α]^20^_D_= - 17 (*c* = 0.29 in CH_2_Cl_2_); ^1^H NMR (400 MHz, CDCl_3_) d 6.28 (ddd, *J*_6–5_ = 10.1 Hz, *J*_6–7_ = 4.7 Hz, *J*_6–7_ = 1.5 Hz, 1H, H_6_), 5.69 (d, *J*_6–5_ = 10.1 Hz, 1H, H_5_), 5.45 (ddd, *J*_2–1_ = 3.1 Hz, *J*_3–2_ = 7.2 Hz, *J*_3–2_ = 10.4 Hz, 1H, H_2_), 4.27 (d, *J*_4–3_ = 6.6 Hz, 1H, H_4_), 2.82 (d, *J*_2–1_ = 3.1 Hz, 1H, H_1_), 2.39 (m, 1H, H_3_), 2.15 (s, 3H, H_13_), 1.91–2.35 (m, 4H, H_3_, H_7_, H_7_, H_8_), 1.27 (s, 3H, H_10_), 0.90 (d, *J*_8–11_ = 6.5 Hz, 3H, H_11_). MS (EI): *m/z* (%) = 220 (28), 124 (96), 109 (72), 95 (29), 43 (100) HRMS (ESI^+^): calcd. for [*M*+Na]^+^ (C_15_H_20_O_5_Na) 303.12029, found 303.12015; elemental analysis calcd (%) C_15_H_20_O_5_: C 64.27, H 7.19, found: C 64.24, H 7.16.

### Refinement

H atoms bonded to C atoms were positioned with idealized geometry and were refined with *U*_iso_(H) = 1.2× *U*_eq_(C)) using a riding model with C—H = 0.96 Å. The H atom bonded to the O atom was located from a difference Fourier syntheses and it was freely refined.

### Crystal data

**Table:**

C_15_H_20_O_5_	*Z* = 2
*M**_r_* = 280.3	*F*(000) = 300
Monoclinic, *P*2_1_	*D*_x_ = 1.288 Mg m^−^^3^
Hall symbol: P 2yb	Mo *K*α radiation, λ = 0.71069 Å
*a* = 10.3312 (10) Å	µ = 0.10 mm^−^^1^
*b* = 7.1692 (8) Å	*T* = 293 K
*c* = 10.8502 (6) Å	Block, colourless
β = 115.958 (5)°	0.48 × 0.42 × 0.30 mm
*V* = 722.56 (12) Å^3^	

### Data collection

**Table:**

Nonius KappaCCD diffractometer	3328 independent reflections
Radiation source: X-ray tube	2774 reflections with *I* > 2σ(*I*)
Graphite monochromator	*R*_int_ = 0.049
CCD, φ and ω frames scans	θ_max_ = 28.1°, θ_min_ = 6.5°
Absorption correction: gaussian (*JANA2006*; Petříček *et al.*, 2006)	*h* = −13→13
*T*_min_ = 0.967, *T*_max_ = 0.972	*k* = −9→9
12282 measured reflections	*l* = −14→14

### Refinement

**Table:**

Refinement on *F*^2^	77 constraints
*R*[*F*^2^ > 2σ(*F*^2^)] = 0.050	H atoms treated by a mixture of independent and constrained refinement
*wR*(*F*^2^) = 0.138	Weighting scheme based on measured s.u.'s *w* = 1/(σ^2^(*I*) + 0.0025000002*I*^2^)
*S* = 1.84	(Δ/σ)_max_ = 0.013
3328 reflections	Δρ_max_ = 0.15 e Å^−^^3^
185 parameters	Δρ_min_ = −0.12 e Å^−^^3^
0 restraints	

### Fractional atomic coordinates and isotropic or equivalent isotropic displacement parameters (Å^2^)

**Table:**

	*x*	*y*	*z*	*U*_iso_*/*U*_eq_	
O4a	−0.24245 (17)	−0.3248 (2)	−0.96277 (13)	0.0469 (6)	
O2	0.13229 (15)	−0.5347 (2)	−0.74186 (16)	0.0540 (6)	
O9	−0.1870 (2)	−0.6248 (2)	−0.95660 (17)	0.0580 (7)	
O4	−0.0236 (2)	0.0757 (2)	−0.7741 (2)	0.0747 (9)	
O12	0.3391 (2)	−0.4418 (4)	−0.5763 (2)	0.0969 (10)	
C1	−0.0910 (2)	−0.4390 (3)	−0.74640 (18)	0.0373 (6)	
C2	0.0592 (2)	−0.3717 (3)	−0.7223 (2)	0.0452 (8)	
C3	0.0495 (3)	−0.2237 (3)	−0.8268 (3)	0.0614 (11)	
C4	−0.0719 (3)	−0.0820 (3)	−0.8628 (3)	0.0554 (10)	
C5	−0.3316 (3)	−0.0382 (3)	−0.9057 (3)	0.0649 (10)	
C6	−0.4264 (3)	−0.0442 (5)	−0.8551 (3)	0.0789 (12)	
C7	−0.4257 (3)	−0.1856 (5)	−0.7544 (3)	0.0787 (13)	
C8	−0.3245 (2)	−0.3495 (4)	−0.7387 (2)	0.0565 (9)	
C9	−0.1760 (2)	−0.4802 (3)	−0.8969 (2)	0.0409 (7)	
C10	−0.0991 (3)	−0.1656 (4)	−0.5957 (2)	0.0558 (9)	
C11	−0.3100 (3)	−0.4779 (6)	−0.6212 (3)	0.0855 (15)	
C12	0.2732 (2)	−0.5534 (3)	−0.6613 (2)	0.0504 (8)	
C13	0.3297 (3)	−0.7288 (4)	−0.6924 (3)	0.0661 (11)	
C8a	−0.1778 (2)	−0.2776 (3)	−0.72788 (18)	0.0400 (7)	
C4a	−0.2064 (2)	−0.1689 (3)	−0.8603 (2)	0.0446 (7)	
H1c5	−0.343345	0.053419	−0.97428	0.0778*	
H1c6	−0.500769	0.048719	−0.885126	0.0947*	
H1c7	−0.521658	−0.231528	−0.781906	0.0945*	
H2c7	−0.398331	−0.127184	−0.666953	0.0945*	
H1c8	−0.365931	−0.42545	−0.819637	0.0678*	
H1c11	−0.262203	−0.590901	−0.625442	0.1026*	
H2c11	−0.254686	−0.416546	−0.535349	0.1026*	
H3c11	−0.403884	−0.507108	−0.628833	0.1026*	
H1c10	−0.162091	−0.070956	−0.590084	0.0669*	
H2c10	−0.070651	−0.247485	−0.518177	0.0669*	
H3c10	−0.015259	−0.108038	−0.595774	0.0669*	
H1c1	−0.078238	−0.541924	−0.68555	0.0448*	
H1c2	0.108852	−0.317946	−0.632628	0.0543*	
H1c13	0.266544	−0.830237	−0.698342	0.0793*	
H2c13	0.335007	−0.715916	−0.778133	0.0793*	
H3c13	0.42399	−0.753581	−0.620827	0.0793*	
H1c3	0.041767	−0.283227	−0.908981	0.0736*	
H2c3	0.139807	−0.159392	−0.795406	0.0736*	
H1c4	−0.099131	−0.040857	−0.955087	0.0665*	
H1	−0.056 (5)	0.173 (7)	−0.818 (4)	0.107 (13)*	

### Atomic displacement parameters (Å^2^)

**Table:**

	*U*^11^	*U*^22^	*U*^33^	*U*^12^	*U*^13^	*U*^23^
O4a	0.0587 (9)	0.0363 (7)	0.0343 (7)	−0.0011 (7)	0.0100 (6)	−0.0009 (5)
O2	0.0389 (8)	0.0493 (8)	0.0649 (9)	0.0047 (7)	0.0145 (7)	−0.0114 (8)
O9	0.0699 (11)	0.0392 (7)	0.0577 (9)	−0.0061 (8)	0.0212 (8)	−0.0126 (7)
O4	0.0777 (13)	0.0319 (8)	0.0961 (15)	−0.0112 (8)	0.0212 (11)	−0.0057 (9)
O12	0.0516 (11)	0.1034 (17)	0.1043 (16)	0.0019 (11)	0.0053 (10)	−0.0446 (15)
C1	0.0363 (9)	0.0341 (8)	0.0368 (9)	−0.0012 (8)	0.0116 (7)	0.0027 (8)
C2	0.0379 (10)	0.0385 (9)	0.0574 (12)	−0.0036 (8)	0.0191 (9)	−0.0110 (9)
C3	0.0679 (15)	0.0409 (10)	0.0914 (18)	−0.0106 (11)	0.0498 (14)	−0.0025 (12)
C4	0.0704 (16)	0.0313 (10)	0.0618 (14)	−0.0047 (10)	0.0264 (12)	0.0031 (9)
C5	0.0615 (15)	0.0462 (11)	0.0603 (14)	0.0115 (12)	0.0022 (12)	0.0025 (11)
C6	0.0530 (15)	0.0752 (17)	0.0814 (18)	0.0256 (15)	0.0043 (13)	−0.0116 (16)
C7	0.0430 (13)	0.105 (2)	0.0816 (18)	0.0144 (15)	0.0214 (12)	−0.0156 (17)
C8	0.0391 (11)	0.0744 (15)	0.0528 (12)	−0.0025 (11)	0.0171 (9)	−0.0064 (12)
C9	0.0448 (10)	0.0342 (9)	0.0414 (9)	−0.0066 (8)	0.0168 (8)	−0.0016 (8)
C10	0.0477 (12)	0.0648 (14)	0.0440 (11)	0.0048 (11)	0.0100 (9)	−0.0161 (11)
C11	0.0634 (18)	0.123 (3)	0.0822 (18)	−0.0143 (19)	0.0428 (15)	0.011 (2)
C12	0.0388 (10)	0.0562 (12)	0.0541 (12)	0.0009 (10)	0.0183 (9)	0.0023 (11)
C13	0.0491 (13)	0.0702 (15)	0.0796 (16)	0.0127 (12)	0.0289 (12)	0.0025 (14)
C8a	0.0353 (9)	0.0430 (9)	0.0351 (9)	0.0008 (8)	0.0091 (7)	−0.0031 (8)
C4a	0.0498 (11)	0.0325 (8)	0.0406 (10)	0.0004 (8)	0.0097 (8)	−0.0019 (8)

### Geometric parameters (Å, º)

**Table:**

O4a—C9	1.340 (2)	C5—H1c5	0.96
O4a—C4a	1.504 (2)	C6—C7	1.488 (5)
O2—C2	1.456 (3)	C6—H1c6	0.96
O2—C12	1.337 (2)	C7—C8	1.532 (4)
O9—C9	1.201 (3)	C7—H1c7	0.96
O4—C4	1.426 (3)	C7—H2c7	0.96
O4—H1	0.83 (5)	C8—C11	1.526 (5)
O12—C12	1.185 (3)	C8—C8a	1.556 (3)
C1—C2	1.535 (3)	C8—H1c8	0.96
C1—C9	1.507 (3)	C10—C8a	1.531 (3)
C1—C8a	1.530 (3)	C10—H1c10	0.96
C1—H1c1	0.96	C10—H2c10	0.96
C2—C3	1.524 (4)	C10—H3c10	0.96
C2—H1c2	0.96	C11—H1c11	0.96
C3—C4	1.526 (4)	C11—H2c11	0.96
C3—H1c3	0.96	C11—H3c11	0.96
C3—H2c3	0.96	C12—C13	1.486 (4)
C4—C4a	1.534 (4)	C13—H1c13	0.96
C4—H1c4	0.96	C13—H2c13	0.96
C5—C6	1.316 (5)	C13—H3c13	0.96
C5—C4a	1.495 (3)	C8a—C4a	1.545 (3)
			
C9—O4a—C4a	108.72 (13)	C7—C8—H1c8	108.87
C2—O2—C12	118.11 (17)	C11—C8—C8a	113.63 (18)
C4—O4—H1	111 (3)	C11—C8—H1c8	105.3
C2—C1—C9	108.2 (2)	C8a—C8—H1c8	106.65
C2—C1—C8a	110.30 (16)	O4a—C9—O9	121.67 (17)
C2—C1—H1c1	107.44	O4a—C9—C1	109.53 (16)
C9—C1—C8a	101.19 (14)	O9—C9—C1	128.80 (17)
C9—C1—H1c1	115.73	C8a—C10—H1c10	109.47
C8a—C1—H1c1	113.8	C8a—C10—H2c10	109.47
O2—C2—C1	105.87 (16)	C8a—C10—H3c10	109.47
O2—C2—C3	108.7 (2)	H1c10—C10—H2c10	109.47
O2—C2—H1c2	112.88	H1c10—C10—H3c10	109.47
C1—C2—C3	111.08 (17)	H2c10—C10—H3c10	109.47
C1—C2—H1c2	110.54	C8—C11—H1c11	109.47
C3—C2—H1c2	107.84	C8—C11—H2c11	109.47
C2—C3—C4	115.7 (3)	C8—C11—H3c11	109.47
C2—C3—H1c3	109.47	H1c11—C11—H2c11	109.47
C2—C3—H2c3	109.47	H1c11—C11—H3c11	109.47
C4—C3—H1c3	109.47	H2c11—C11—H3c11	109.47
C4—C3—H2c3	109.47	O2—C12—O12	122.1 (2)
H1c3—C3—H2c3	102.43	O2—C12—C13	111.11 (19)
O4—C4—C3	110.43 (19)	O12—C12—C13	126.8 (2)
O4—C4—C4a	111.3 (3)	C12—C13—H1c13	109.47
O4—C4—H1c4	108.45	C12—C13—H2c13	109.47
C3—C4—C4a	111.97 (19)	C12—C13—H3c13	109.47
C3—C4—H1c4	107.71	H1c13—C13—H2c13	109.47
C4a—C4—H1c4	106.8	H1c13—C13—H3c13	109.47
C6—C5—C4a	122.5 (3)	H2c13—C13—H3c13	109.47
C6—C5—H1c5	118.76	C1—C8a—C8	110.28 (18)
C4a—C5—H1c5	118.76	C1—C8a—C10	114.76 (15)
C5—C6—C7	124.3 (3)	C1—C8a—C4a	98.29 (18)
C5—C6—H1c6	117.87	C8—C8a—C10	110.2 (2)
C7—C6—H1c6	117.87	C8—C8a—C4a	108.26 (15)
C6—C7—C8	112.8 (3)	C10—C8a—C4a	114.46 (17)
C6—C7—H1c7	109.47	O4a—C4a—C4	102.8 (2)
C6—C7—H2c7	109.47	O4a—C4a—C5	108.89 (15)
C8—C7—H1c7	109.47	O4a—C4a—C8a	101.37 (14)
C8—C7—H2c7	109.47	C4—C4a—C5	113.57 (19)
H1c7—C7—H2c7	105.88	C4—C4a—C8a	114.52 (16)
C7—C8—C11	111.6 (3)	C5—C4a—C8a	114.1 (2)
C7—C8—C8a	110.4 (2)		

### Hydrogen-bond geometry (Å, º)

**Table:**

*D*—H···*A*	*D*—H	H···*A*	*D*···*A*	*D*—H···*A*
C8—H1*c*8···C9	0.96	2.48	2.913 (4)	107.28
C10—H3*c*10···O4	0.96	2.31	2.946 (4)	122.91
C2—H1*c*2···O12	0.96	2.36	2.668 (3)	98.20
O4—H1···O9^i^	0.83 (5)	2.10 (4)	2.907 (2)	165 (4)

Symmetry code: (i) *x*, *y*+1, *z*.
